# High Prevalence of Bovine Cardiac Cysticercosis in Upper Egypt: An Epidemiological and Histopathological Study

**DOI:** 10.3390/ani14010158

**Published:** 2024-01-03

**Authors:** Fatma A. S. Anwar, Eman A. Negm, Maha Abdelhaseib, Fatma M. Abdel-maksoud, Ahmed A. Mohammed, Sara Abdel-Aal Mohamed, Ahmed Gareh, Nady Khairy Elbarbary, Manal F. El-khadragy, Ehssan Ahmed Hassan, Ehab Kotb Elmahallawy

**Affiliations:** 1 Department of Zoology, Faculty of Science, Assiut University, Assiut 71526, Egypt; fatma.anwar@science.aun.edu.eg; 2 Department of Physiology, Faculty of Veterinary Medicine, Assiut University, Assiut 71526, Egypt; emannegm@vet.aun.edu.eg; 3 Department of Animal Physiology and Biochemistry, School of Veterinary Medicine, Badr University in Assiut, New Nasser City, Assiut 11829, Egypt; 4 Department of Food Hygiene, Faculty of Veterinary Medicine, Assiut University, Assiut 71526, Egypt; maha83abdelhaseib@gmail.com; 5 Department of Anatomy and Embryology, Faculty of Veterinary Medicine, Assiut University, Assiut 71526, Egypt; fatma.abdelmaksoud@vet.au.edu.eg; 6 Department of Anatomy and Embryology, Faculty of Veterinary Medicine, Sphinx University, Assiut 71515, Egypt; 7 Department of Animal and Poultry Behavior and Management, Faculty of Veterinary Medicine, Assiut University, Assiut 71526, Egypt; 8 Department of Parasitology, Faculty of Veterinary Medicine, Assiut University, Assiut 71526, Egypt; 9 Department of Parasitology, Faculty of Veterinary Medicine, Aswan University, Aswan 24101, Egypt; ahmedgareh86@gmail.com; 10 Department of Food Hygiene, Faculty of Veterinary Medicine, Aswan University, Aswan 81528, Egypt; nadyvet82@yahoo.com; 11 Department of Biology, College of Science, Princess Nourah bint Abdulrahman University, P.O. Box 84428, Riyadh 11671, Saudi Arabia; 12 Department of Biology, College of Science and Humanities in Alkharj, Prince Sattam Bin Abdulaziz University, Alkharj 11942, Saudi Arabia; 13 Department of Zoology, Faculty of Science, Suez Canal University, El-Sheikh Zayed, Ismailia 41522, Egypt; 14 Departamento de Sanidad Animal, Grupo de Investigación en Sanidad Animal y Zoonosis (GISAZ), Facultad de Veterinaria, Universidad de Córdoba, E-14071 Córdoba, Spain; 15 Department of Zoonoses, Faculty of Veterinary Medicine, Sohag University, Sohag 82524, Egypt

**Keywords:** *Cysticercus bovis*, cattle, prevalence, physiological parameters, meat inspection, histopathological

## Abstract

**Simple Summary:**

Bovine cysticercosis is considered one of the major parasitic zoonoses that carry an important food safety concern. The disease can lead to a range of symptoms and health problems based on the location and number of cysts. Moreover, bovine cysticercosis can cause considerable economic losses in livestock production. Clearly, there is an urgent need for a practical and sensitive method for the detection of infected carcasses. Little information is known about the prevalence of cardiac cysticercosis in bovines from Upper Egypt and the potential use of histopathological and immunohistochemical methods in the localization of the pathogen. The present study used a set of morphological, morphometric, histopathological and immunohistochemical techniques to investigate the distribution of the cardiac cysticercosis in cattle from Upper Egypt. Furthermore, the measurement of various biochemical markers was performed to identify their possible association with the infection.

**Abstract:**

Bovine cysticercosis is categorized as a serious parasitic zoonotic infestation. The infection is mainly caused by the tapeworm *Taenia saginata*, which infects cattle and humans. The larval stage, *Cysticercus bovis (C. bovis)*, is found in the skeletal and cardiac muscles of infected cattle. Despite its potential public health concern, few studies have been conducted on cardiac cysticercosis in Upper Egypt. This study investigates the prevalence, epidemiology, and impact of cardiac cysticercosis in Upper Egypt, emphasizing how histopathological changes in cardiac muscle and physiological parameters might be associated with the infection. From December 2022 to October 2023, a total of 941 animals from Assiut province, Upper Egypt, were slaughtered and their cardiac muscles were examined for the presence of *C. bovis*. Cysts were classified as viable or degenerated through macroscopic inspection. The overall prevalence of *C. bovis* infected hearts made up 10.8% of the total examined. The highest prevalence rate was in the summer season followed by spring; winter had the lowest infections. The histopathological examination of infected tissues revealed immune cell infiltration around Cysticercus-infected areas. Additionally, Bax immunostaining demonstrated the apoptotic effect of cysticercosis. Regarding the measured physiological parameters, there were non-significant changes in plasma levels of total protein and albumin in cattle infected with cysticercosis compared with control animals. Moreover, there was a significant decrease in total antioxidant capacity (TAC) combined with a significant increase in lipid peroxide (Malondialdehyde) (MDA), troponin T, and lactate dehydrogenase (LDH) activity in infected animals. The present work documented a set of epidemiological and pathological findings, revealing that *C. bovis* is a potentially harmful parasite and can cause significant health problems in both cattle and humans.

## 1. Introduction

Bovine cysticercosis is a parasitic disease caused by the larval stage (*Cysticercus bovis*) of the cestode *Taenia saginata*. Cattle serve as the intermediate host in the two-host life cycle, while humans represent the definitive hosts. Globally, around 50 million human cases were reported resulting in ~50 thousand fatalities annually [1,2]. Generally, humans get infected by consuming raw or undercooked meat containing infective *cysticerci* that develop into full grown tapeworms three months later. Taken into account, human taeniasis is asymptomatic or presented through mild gastrointestinal symptoms, nausea, vomiting, abdominal discomfort, flatulence, and diarrhea. However, other complications such as weight loss and vitamin deficiency, excessive loss of appetite, weakness, and intestinal blockage might take place. Moreover, psychological stress caused by the active migration of proglottids from the anus and the length of the tapeworm has been reported [3]. Importantly, infected people can shed millions of eggs daily into the environment; these eggs can survive for up to seven months and could be transmitted to the intermediate host [4]. Commonly, cattle become infected through the ingestion of grass, grains, or water contaminated with eggs [4,5]. Then, the hatching of ingested eggs occur within the small intestine to produce oncospheres that penetrate the intestinal wall and migrate through the bloodstream to the muscles where they may grow into infectious cysts. Humans become infected by ingesting raw or inadequately cooked meat containing *C. bovis*. Typically, the masseter muscles, heart, tongue, diaphragm, and other skeletal muscles contain live cysticerci, while there are rarely found in fat or visceral organs. Cysticerci undergo degeneration after a period of time that can range from weeks to years followed by calcification [6]. In cattle, natural infections are normally asymptomatic. However, heavy infestation by larvae may cause myocarditis or heart failure [7].

Although the epidemiological distribution of bovine cysticercosis is worldwide, the incidence of the disease in developing countries is higher than developed countries. Prevalence rates of 30–80% have been reported in some East African countries [7,8]. In the meat industry, economic losses are significantly associated with infection by these parasites. Carcasses must be completely condemned they have heavy infestations. However, light infection or localized cysticercosis leads to the condemnation of the infected parts. Additionally, the carcass must be kept in freezing storage fir up to 3 weeks to inactivate the parasites [9]. In this regard, the projected cost of the handling, transporting, and refrigerating of infected carcasses in the United Kingdom was estimated to exceed GBP 4.0 million yearly [10]. Similarly, bovine cysticercosis causes significant losses in Africa that have been estimated to be approximately USD 1.8 billion annually [9].

Regarding the diagnosis of the infection, a visual examination of the cysticerci during the post-mortem examination of carcasses is typically employed for the detection of the parasites. Nevertheless, traditional methods have major drawbacks and limited diagnostic capability [1,11]. Moreover, standard inspection techniques are ineffective for detecting bovine cysticercosis at low concentrations. In Egypt, the diagnosis of detecting of bovine cysticercosis is totally based on the postmortem examination of the carcass. In the same context, lesions in the heart or masseter muscles that include entirely transparent, cheesy, or calcified cysts are thought to be *Cysticercus* cysts [5,6]. Taken into account, the presence of abscesses, granulomas, or parasites such as *Sarcocystis* spp. associated with eosinophilic myositis may develop similar macroscopic lesions [8]. The developed immunohistochemical test provides an inexpensive, reliable means of diagnosis in tissue sections and differentiating them from normal tissues and other pathogenic lesions [10,12]. Despite its zoonotic potential, few studies have been conducted to investigate the current status of bovine cyctsercosis in Upper Egypt. Furthermore, limited information is reported about the morphology and morphometric measurements of cysticerci in this area of Egypt. This study aimed to determine the prevalence of *C. bovis* in cattle slaughtered in Assiut province in Upper Egypt using a set of different diagnostic serological and immunohistochemical techniques. The work also included a descriptive histopathological study of viable and calcified cysts by using light and scanning electron microscopes. Furthermore, biochemical tests and immunohistochemistry (IHC) were employed for confirmation, which might represent a step towards establishing a more dependable method for identifying the effects of *C. bovis* on bovine tissue.

## 2. Materials and Methods

### 2.1. Study Area, Period and Design

This study included a cross-sectional study conducted from December 2022 to October 2022 to determine the prevalence rate of *C. bovis* in Assiut province located in Upper Egypt.

### 2.2. Animals

A total of 941 animals admitted to various slaughterhouses in Assiut province, including *(n* = 852) native bred cattle and (*n* = 89) cases from other breeds (Holstein and Brazilian breeds), were included in the present study. Antemortem examinations of the animals were performed for the evaluation of their general conditions. All data, including sex, breed, and season of sample collection, were recorded routinely. Carcasses were subjected to complete postmortem examination via visual inspection, palpation, and multiple incisions of the organs. The present study was focused on the examination of hearts found to be positive with *Cysticercus bovis*. The samples used for this study were selected from the condemned infected heart specimen after routine examinations in Assiut slaughterhouse. The ethics committee of the Faculty of Veterinary Medicine, Assiut University, Egypt, approved this study (no. 06/2022/0014). Hearts were also collected to perform the morphological and ultrastructural identification of the cyst.

### 2.3. Blood Collection

Blood samples were collected from all live cases studies through the study period and then were placed in tubes containing EDTA. The positive and control samples were effectively recognizable after slaughter, with five samples from infected animals and five from healthy ones. Then, samples were centrifuged at 3000 rpm for 10 min and plasma was obtained and stored at −20 °C.

### 2.4. Biochemical Determinations

Plasma of negative and positive *C. bovis* cattle (*n* = 5 each) was used for the estimation of LDH activity spectrophotometrically using reagent kits purchased from Human Gesell Schaft fur Biochemical und Diagnostic mbH, Germany, while plasma total protein and albumin levels were determined using reagent kits purchased from DiaSys Diagnostic System GmbH, Germany. Plasma levels of TAC and MDA were estimated using reagent kits obtained from Biodiagnostic, Giza, Egypt.

Plasma cardiac troponin T (cTnT) (a protein integral to the contraction of heart muscles) levels were measured using the ELISA technique (Dynatech microplate reader model MR 5000, Chantilly, VA, USA) and reagent kits (No. SG-10127) purchased from SinoGeneClon Biotech Co., Ltd., No. 9 BoYuan Road, Yu Hang District, Hangzhou, China.

### 2.5. Cyst Collection and Preservation

Cysticerci were collected and classified into degenerated and viable cysts after macroscopic examination. Cysts that were filled with translucent fluid were considered viable, while cysts that were empty or those with solid contents were considered degenerating or non-viable [13]. Cysts were then washed using PBS to remove debris and prepared specimens were carefully subjected to horizontal incision and preserved in 3% glutaraldehyde for further examination using a scanning electron microscope (SEM). For histopathological examination, cysts were preserved in a neutral buffer formalin, 10%. All samples were transported in sterile containers directly to Assiut University, Faculty of Veterinary Medicine, Parasitology Department.

### 2.6. Scanning Electron Microscopical Examination of the Cyst

The incised specimens were dehydrated through graded series of ethanol, critical-point-dried, coated with gold, and examined at the Electron Microscopy Unit at Assiut University.

### 2.7. Histopathological Examination of the Cyst and the Surrounding Heart Tissue

The cysts and surrounding heart tissue from five infected animals were fixed in neutral-buffered formalin, processed in serial concentrations of ethyl alcohol, cleared in xylene, and then paraffin-embedded. The histological sections were stained using hematoxylin and eosin (H & E) and photographed using a Canon digital camera (Canon Powershot A95) mounted on a Leitz Dialux 20 microscope (Wetzlar, Germany).

### 2.8. Semi-Thin Sections and Transmission Electron Microscopical Examination of the Cyst and the Surrounding Heart Muscle

Small specimens from the normal and cyst-infected hearts were preserved through immersion in a mixture of 3% PFA–glutaraldehyde in 0.1 mol/L Na-cacodylate buffer at a pH of 7.2 for 48 h at 4 °C. The samples were washed with the same buffer and then postfixed in 1% osmic acid in 0.1 M Na-cacodylate buffer for 2 h at room temperature (RT). The specimens were dehydrated in an ascending series of ethanol and embedded in an Araldite–Epon mixture. The sections were cut at a thickness of 1 μm and stained with 1% toluidine blue in line with our previous study [14]. The stained sections were examined using the Leitz Dialux 20 microscope and the photos of these sections were captured using a Canon digital camera (Canon Power shot A 95). Ultrathin sections were cut using an Ultrotom VRV (LKB Bromma, Oberkochen, Germany). Subsequently, the sections (70 nm) were stained with uranyl acetate and lead citrate and then examined using the JEOL 100CX II transmission electron microscope (TEM) (JEOL, Tokyo, Japan) at the Electron Microscopy Unit at Assiut University.

### 2.9. Immunohistochemical Analysis

Immunohistochemistry was performed as described previously [15,16,17,18]. The paraffin sections were deparaffinized and hydrated and then washed with 1 mML of PBS (three times, 5 min each). To unmask the antigen epitopes, antigen retrieval was carried out using 1 mML of sodium citrate buffer solution (pH 6). The sections were washed with PBS (pH 7.4) for 15 min. After blocking the endogenous peroxidase activity with 3% H_2_O_2_, sections were washed with PBS (3 × 5 min) and then were incubated overnight at 4 °C with Bax (Rabbit Polyclonal-Polyscience Lab, Seoul, Korea).

### 2.10. Statistical Analysis

Prevalence data were verified, coded, and analyzed using IBM-SPSS 24.0 (IBM-SPSS Inc., Chicago, IL, USA). Regarding the descriptive statistics, frequency and percentages were calculated and the Chi-square test/Fisher’s exact test were used to compare the differences in frequency between groups as appropriate. Measurements of biochemical data were represented as mean ± standard error of the mean (SEM). The results were then analyzed utilizing Student’s *t*-test analysis using Prism software (version 8.0.1; GraphPad Software, Inc., San Diego, CA, USA). A *p*-value of <0.05 was considered significant.

## 3. Results

### 3.1. Prevalence of Cardiac Cysticercosis

The prevalence rate of *C. bovis* among examined hearts was 10.8% from the total examined (102 out of 941). The collected (*n* = 102) cysts were of different sizes and ages (including viable and degenerated ones). In relation to breeds, bovine cysticercosis was reported among the native cattle breeds and not recorded in the imported cattle breeds (Holstein and Brazilian breeds). All the infections were only recorded among male animals aged from 1.5 to 2 years old (Table 1). Concerning season, the prevalence rate was highly significant with regard to the summer season (79 out of the 102 cases) followed by spring, while the lowest infection rate was in winter.

### 3.2. Gross and SEM Studies

**Figure 1:**
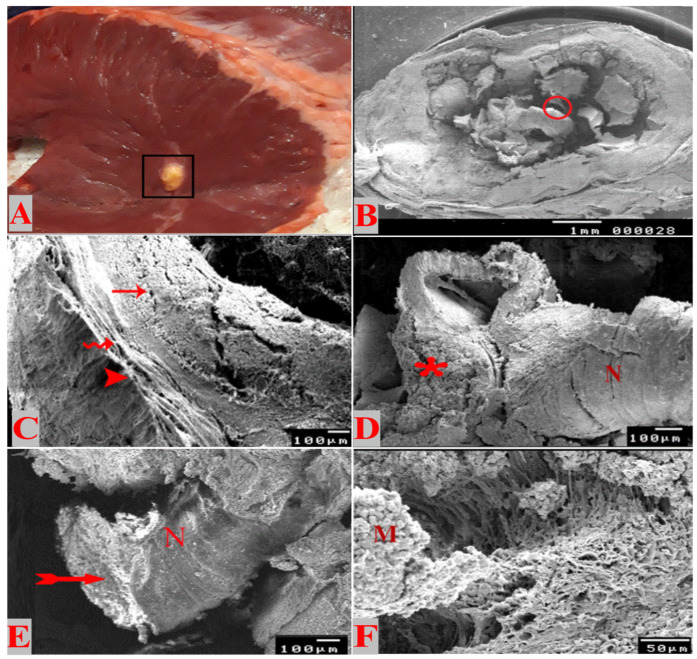
Gross and SEM photographs of *Cysticercus bovis* affecting cattle heart. (**A**) Calcified non-viable cyst (square), (**B**) SEM photograph of the invaginated scolex (circle), (**C**) calcified cyst at a higher magnification of the bladder showing heart (head arrow), attachment of microtriches with heart muscle (wavy arrow), and thick fibrous layer (thin arrow), (**D**) higher magnification of the neck (N) and evagination site (asterisk), (**E**) calcified cyst showing calcified parts of the neck (N) and calcified parts (arrow), and (**F**) the attachment of the cyst with the heart muscle through microtriches (M).

The collected positive hearts were carefully examined and a total of 102 cysticerci were dissected and their size ranged from 0.5 to 1.4 cm. As depicted in Figure 1A, the macroscopic examination of cysticerci revealed the presence of two viable cysticerci and their wall was relatively thinner; the cyst was containing translucent to faint white material and a visible invaginated scolex. The two viable cysticerci were found in the summer season from two different cattle that were apparently healthy. The calcified ones (comprising the other 100 samples) were in different stages of degeneration and their walls were hard, containing solid materials and a salty exudate (Figure 1B). The SEM of the incised susceptible, viable or recently degenerated cysts showed the presence of a chamber containing the invaginated scolex with tegumental fold at the bladder opening (Figure 1C). The cyst was surrounded by a relatively thin fibrous layer separating it from the surrounding cardiac muscle (Figure 1D). The invaginated scolex was visible from the neck and septa and the site of the beginning of proglottide generation (Figure 1E). On the other hand, the degenerating cysticerci showed the presence of several calcified fragments and parts of the degenerated neck within the cavity of the cyst. The fibrous layer was relatively thick, while the tegumental fold was not noticed, nor was the bladder opening (Figure 1F). The site of attachment between the bladder and the muscle tissue showed the strong attachment between microtriches and muscle fibers (Figure 1F). The fibrous layer was relatively thicker than that of viable cysts (Figure 1D), while the tegumental fold was not noticed, nor were the bladder opening and site of proglottide generation (Figure 1F).

### 3.3. Histopathological and Semi-Thin Sections of Cysticerci

**Figure 2:**
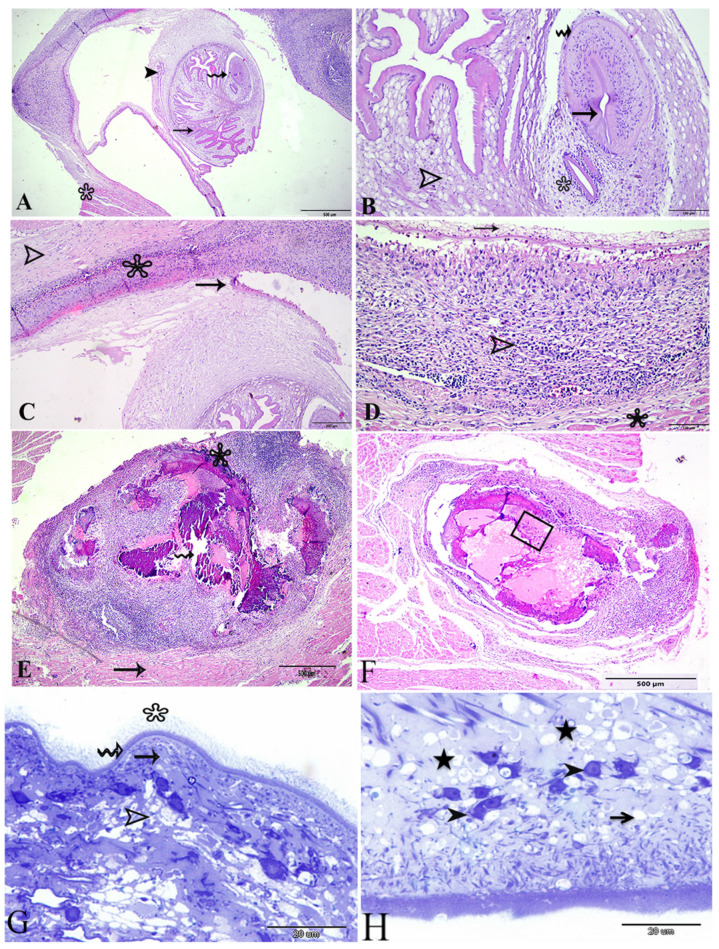
Photomicrograph sections of *Cysticercus bovis* affecting cattle heart stained with H&E showing (**A**) viable or young Cysticercus, convoluted spiral canal (thin arrow), invaginated scolex (wavy arrow), vestibule (head arrow), and thin wall of the cyst attached to heart muscles (asterisk); (**B**) higher magnification of the cyst showing the reticular parenchymatous layer (head arrow), sucker (asterisk), the scolex (wavy arrow), and sucker (thin arrow); (**C**) inflammatory reaction (asterisk) separating the cyst wall (thin arrow) from the heart muscle (arrow head); (**D**) mixed inflammatory cells (minor) (head arrow) between the heart muscle (star) and cyst fibrous layer (thin arrow); (**E**) calcified cyst formed through a central cavity necrotic core (wavy arrow) and an intense pyogranulomatous reaction (major) (asterisk) within the heart muscle (thin arrow); (**F**) calcified cyst with aggregation of calcareous corpuscles (square); (**G**) photomicrograph of a semi-thin section of *C. bovis* stained with toluidine blue showing the *C. bovis* layers and the three distinctive layers of the Cysticercus, with the outer tegumental layer (wavy arrow) having straight filamentous microtriches (asterisk), the middle cellular layer (thin arrow) containing tegumental cells (spindle shape), and the innermost reticular layer (head arrow); and (**H**) translucent vesicles (thin arrow), tegumental cells (head arrows), and calcareous corpuscles (star).

**Figure 3:**
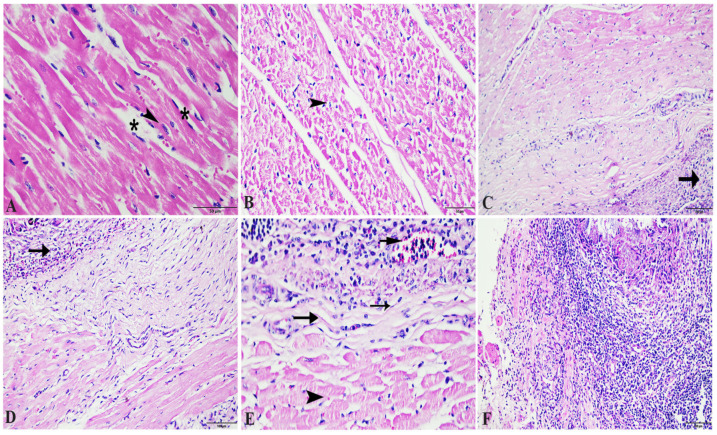
Photomicrographs of the bovine cardiac muscle stained with H & E showing (**A**) the normal appearance of the cardiac muscle fibers with normal striated and branching cardiac cells with the nucleus (arrowhead) as well as perinuclear spaces (asterisk) and (**B**–**F**) abnormal degenerated cardiac muscle fibers (arrow heads) with heavy inflammatory cells and fibroblast infiltrations (arrows).

Histological sections of the viable *Cysticercus* showed that the whole cyst was surrounded by a thin fibrous layer that lies between the cyst and the cardiac muscles (Figure 2E). There was a cavity around the cyst; the bladder wall was thin surrounding the invaginated scolex (Figure 3). The cyst had a bladder opening from which the parasite invaginated. The invagination of the scolex and the neck formed a spiral canal leading to the deepest part of the cyst where the scolex invaginated inside (Figure 2A). This canal was markedly corrugated and lined with the tegumental layers that were analogues of the future adult cestode; the suckers were located toward the blind side of the cyst (Figure 2B). The folded membrane of the spiral canal was lined with a highly eosinophilic homogenous cuticle that was markedly branched. It was lined with the three layers of the cuticular layer, followed by the middle cellular layer, and then by the reticular layer; the parenchyma was thicker around the scolex and suckers and the surface of the parenchymatous portion facing the bladder cavity were lined with a fibrous tissue (receptaculum) (Figure 2A,B).

The fibrous layer surrounding the cyst was thin followed by a cellular layer (where intense inflammatory reaction occurs) between the cardiac muscle and the thin fibrous layer of the cyst. This cellular layer, together with the fibrous layer (host originated), represented the immune response of the host where many inflammatory cells were accumulated, including ellipsoidal cells and multi-nucleated giant cells (Figure 2D). This formal layer was then followed by the cardiac muscles.

On other hand, the histological examination of the degenerated cysticerci showed the impossibility of noticing the scolex or any parts of the parasite tegumental layers. The cyst had a central necrotic area with caseation, extensive mineralization (calcification), and foci were found inside the cyst (Figure 2B). There was a layer of giant cells between the mineralized area and the surrounding cellular layer (Figure 2E) that comprised an extensive pyogranulomatous reaction with mixed inflammatory cells. Another section of degenerated cyst showed an aggregation of diagnostic calcareous corpuscles in the necrotic core (Figure 2F). Semi-thin sections of the susceptible, viable, or recently degenerated cysticerci showed the parasite cuticle had three distinct layers. The first layer was the microtriches that were carried by dense apical plasma membrane and appeared to be different in length with regard to the facing side; they appeared longer in the host side than the bladder side (Figure 2G,H). The second layer, following microtriches, was the cuticular- (vesicular layer) dense cytoplasmic layer that contained electron-dense granules and many translucent vesicles (Figure 2G,H). The third layer was the sub-tegumental layer containing distinct tegumental cells that appeared spindle-shaped with large nuclei containing dense chromatin bodies and perinuclear cytoplasms; it was connected to the distal cytoplasm through cytoplasmic extensions (Figure 2G,H). In addition, calcareous corpuscles were noticed in this layer. The following layer was the muscular layer that contained circular and longitudinal muscle bundles and numerous muscle fibers (Figure 2H).

### 3.4. Histopathological, Transmission Electron Microscopic and Immunohistochemical Analysis of the Cardiac Muscle

**Figure 4:**
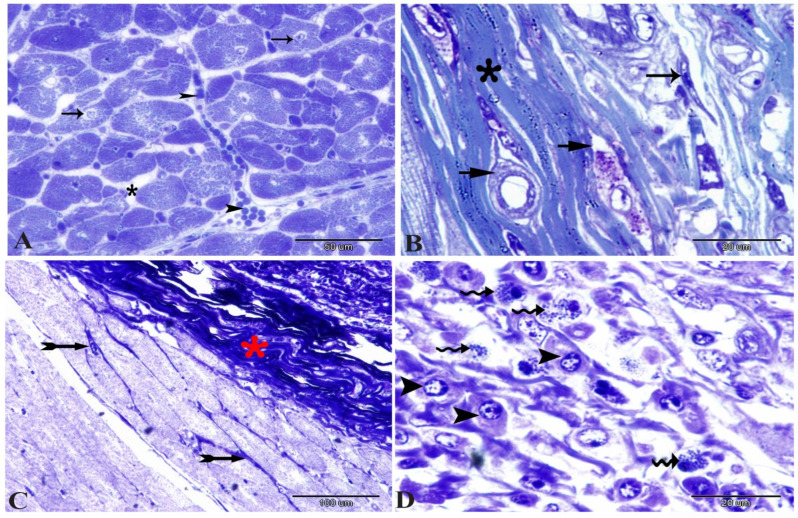
Photomicrographs of a semi-thin section of the cardiac muscle stained with toluidine blue showing (**A**) cross-section of the normal cardiac muscle fibers with central nuclei (arrows) and scanty connective tissue (asterisk) between the cardiac muscle fibers containing secretory granules (arrow heads); (**B**) intense collagen fiber (asterisk) disposition between the cardiac muscle fibers containing dilated and congested blood capillaries (short arrows) and fibroblast (arrow) infiltrations; (**C**) increase in the interstitial connective tissue (arrows), especially near the cyst (note the dense collagen fibers (asterisk)); (**D**) localization of mast (wavy arrow) and plasma cells (arrow heads) within the infiltration that surround the parasite.

**Figure 5:**
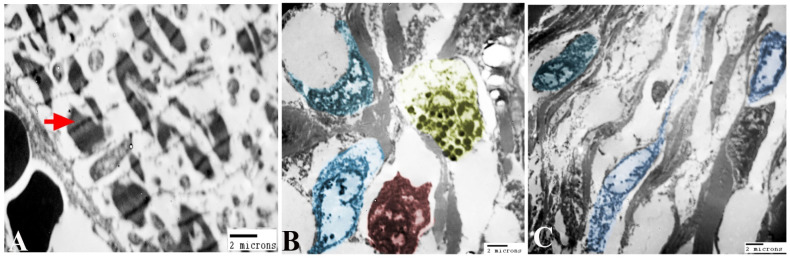
Photomicrographs of a TEM showing (**A**) degeneration of cardiac muscle fibers and unclear banding of sarcomeres (arrow) and disorganization of mitochondria (arrows) and (**B**,**C**) the wall of the cyst consists of mast (yellow), macrophage (red), and many fibroblast (blue) cells.

**Figure 6:**
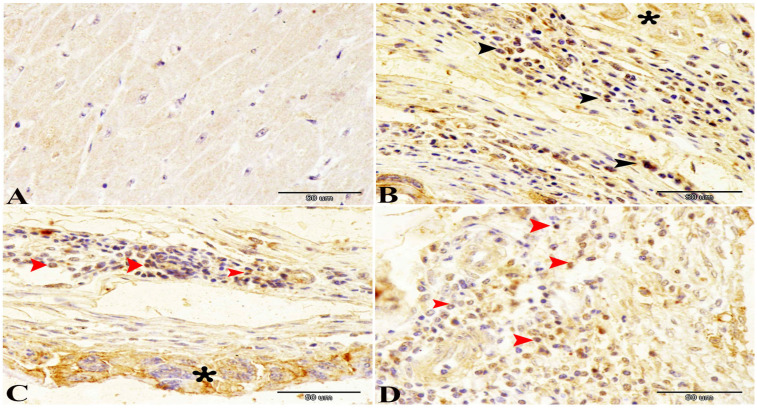
Photomicrographs of Bax immunohistochemical staining showing (**A**) negative reaction in the cardiac muscle of the control group and (**B**–**D**) strong positive staining within the cardiac muscle (asterisk) that surrounds the cyst and in the inflammatory cells (arrow heads) infiltrating the cyst.

Normally, the cardiac muscle is striated, branched, and arranged in densely packed myofibrils as well as separated by a narrow interstitial space that is occupied by rare connective tissue (Figure 3A and Figure 4A). The infected heart muscle showed degenerative changes including a loss of striations, the fragmentation of myofibrils, an increase of the perinuclear spaces, and disorganization of the muscle fibers, especially around the cyst (Figure 3). Furthermore, the infected cardiac muscle showed intense collagen fiber disposition between the cardiac muscle fibers that contained dilated and congested blood capillaries and fibroblast infiltrations (Figure 4B). There was a storm of immune cell infiltration, including the lymphocytes, plasma, macrophages, and mast cells between the muscle fibers and within the cyst (Figure 4D). Additionally, much fibroblast infiltration led to intense deposition of the collagen fibers in the interstitium and around the cyst (Figure 4C). At the ultrastructural level, the degenerative cardiac muscle fibers showed unclear banding of the sarcomeres and disorganization of the mitochondria. The wall of the cyst consisted of mast cells, macrophages, and many fibroblast cells (Figure 5). The Bax immunohistochemical staining showed a negative reaction in the cardiac muscle of the control group but strong positive staining within the cardiac muscle that surrounded the cyst as well as in the inflammatory cells infiltrating the cyst (Figure 6).

### 3.5. Biochemical Parameters of Positive and Negative Cases

Effects of *C. bovis* infection on the levels of plasma total proteins, albumin, MDA, TAC, troponin T, and LDH are presented in Table 2. There were non-significant changes in the levels of total proteins and albumin between the control and cattle infected with cysticercosis (*p* > 0.05). However, there was a significant loss of levels of TAC in cattle infected with cysticercosis compared with the control (*p* < 0.05). While there was a significant increase in levels of MDA, troponin T, and LDH in cattle infected with cysticercosis compared with the control (*p* < 0.05).

## 4. Discussion

Despite causing a considerable economic loss in the meat industry due to the condemnation of meat or carcass downgrading in slaughterhouses, limited data are available about the prevalence and risk factors associated with bovine cysticercosis in developing countries. Clearly, providing daily disease records of all slaughtered animals is mandatory [19]. Our study reports bovine cardiac cysticercosis with a prevalence rate of 10.8% among the examined animals. The prevalence of cysticercosis varies between the different regions of Egypt and some areas still have a higher prevalence [13]. Previous works [13,20] report a prevalence rate of 4.2% and 3.63% in cattle in Egypt, which are less than the current results. However, another previous study [21] agrees with the present results and reports a prevalence rate of 7.5% in Aswan city (Upper Egypt). *C. bovis* is considered endemic in many parts of Africa, particularly in areas with poor sanitation and inadequate meat inspection practices. The prevalence in cattle has been reported to be as high as 50% and 46.7% in Africa and Asia, respectively [22,23]. Nevertheless, *C. bovis* may be underestimated in many regions due to inadequate meat inspection practices, a lack of awareness among consumers and producers, and limited access to diagnostic tools [24]. The prevalence of *C. bovis* in slaughtered cattle has been reported to be 21.8%, 22.6%, 27.3%, and 26.2% in Nigeria, Uganda, Ethiopia, and Tanzania, respectively [2,25,26,27]. However, it should be underlined that the prevalence of *C. bovis* might vary in different regions of the world depending on various factors such as geographic location, cultural practices, and animal husbandry practices. Importantly, the prevalence of *C. bovis* in African countries might be higher than reported as many slaughterhouses lack proper meat inspection and hygiene practices. Moreover, studies on the prevalence of *C. bovis* often use different diagnostic methods and sampling techniques, making comparisons between studies difficult [26,27]. The studied area is considered one of Upper Egypt’s governorates that have many rural areas where native breeds of cattle are reared. Before entering the slaughterhouses, most animals have been grazing in surrounding pastures, while imported breeds are reared through intensive farming breeding systems. It is important to note that the prevalence of cysticercosis may be influenced by various factors, including climate and animal husbandry practices [28]. Regarding seasonal variation, there is limited information available on the seasonal prevalence of *C. bovis* in Egypt. This study reveals a higher prevalence of *C. bovis* during hot weather (June–August) that might be attributed to the length of the grazing season and the proportion of grazed grass in the diet [28]. Taken into account, the onset of cardiac lesions after the ingestion of eggs on the pasture can be linked with the latency time. Furthermore, it is important to note that the exact reasons for the higher prevalence of bovine cardiac cysticercosis in the summer season may vary and could be influenced by factors such as temperature, farm management practices, and sampling methods [29]. There are many contradictions in seasonal variation between the previous studies [19]. The highest prevalence rate has been detected during summer and declines in spring and autumn, while the lowest prevalence rate is in winter and this result is consistent with our results [19]. It is important to note that the exact reasons for the higher prevalence of bovine cardiac cysticercosis in the summer season may vary and could be influenced by factors such as temperature, farm management practices, and sampling methods [29]. Other studies in Egypt report that winter season has the highest infection rate [30]. The variations in infection rate might be due to differences in temperature degree and humidity. Moreover, the decline in prevalence in urban cities can be attributed to the implementation of control measures such as the prohibition of feeding raw meat to animals.

Meat inspection plays a crucial role in preventing the transmission of *C. bovis* to humans through contaminated meat. In Egypt, the government has implemented strict regulations regarding meat inspection at different levels, including at the abattoirs and at the retail markets [13]. For the precise diagnosis of *C. bovis* through meat inspection, the viable cysticerci are more easily identified and confirmed by their morphological characteristics such as the slightly transparent smooth wall, invaginated scolex, and the translucent fluid filling it. These characteristics can be confirmed using SEM, TEM, and histopathology, while the judgment on the calcified (mineralized) cysts in the heart that are to be calcified as cases of *C. bovis* through macroscopic examination may overestimate the results of the prevalence rate of bovine cysticercosis due to confusion with other lesions. Moreover, the specific diagnosis in slaughterhouses during postmortem inspection is very difficult and meat inspection has a low sensitivity [1]. Degenerating cysts are more frequently found during postmortem inspection than the viable cysts [12]. In the present study, degenerating cysts are the most prevalent (100 out of 102) among the encountered cysts. Taken into account, the demonstration of a scolex in a degenerate lesion is diagnostic of tapeworm infections; however, this is not always possible. In this respect, SEM could help in the visualization of some remnants of the parasite.

Bovine cysticercosis could be misdiagnosed with other macroscopic lesions, such as actinobacillosis [12] and sarcocystosis, which can be found in cattle carcasses [31]. Molecular diagnosis can be performed to discriminate between these diseases, but the expensive cost and limited financial resources remain a major barrier to performing these techniques at a large scale, particularly in developing countries. Clearly, confirmatory tests should be performed for certain diagnoses using histopathology and TEM which are helpful in the identification of the causative agent through the scolex, sucker, and tegumental layers. In the case of mineralized cysts, where there is no way to visualize the scolex or any tegumental parts, histopathological sections can aid in the diagnosis through the presence of granulomatous reactions containing multinucleated giant cells that indicate inflammation. Different pathogens contribute to the granuloma formation that is characterized by focal necrosis and calcification as well as mixed inflammatory cells [32,33]. However, in the current study, all inflammatory and the multinucleated giant cells are detected, suggesting cystic lesion and cysticercosis rather than other pathogens. The histopathological detection of bovine cysticercosis in cases of calcified cysts is undertaken through the detection of some microscopic, characteristic structures such as the calcareous corpuscles that are represented by small elliptical blank spaces [1]. Semi-thin sections of the viable cysts help in the identification of the parasitic tegumental layers that represent the basic components of other larval cestodes and the future adult cestode. The basic layers’ components are microtriches, the vesicular (cellular) layer, and the reticular layer. As observed in the current study, the first layer is the microtrichia [34] and their function includes absorption, transportation, protection, and anchorage [35]. The second layer is the vesicular layer, which has the common characteristics of the distal cytoplasm layer of the cestode in addition to many dense inclusion bodies and lucent vacuoles, suggesting their secretory, excretory, or pinocytotic functions [34]. The tegumental layer contains many spindle-shaped tegument cells, suggesting their neurosecretory nature [36] and, possibly, that they absorb nutrients from the host in the described cysticerci [34]. The parenchyma, with its numerous inclusions, has a storage function and muscular contractions are probably responsible for the movement of fluids within the interstices between the parenchymal tissues. All the previous data help in the confirmation of the diagnosis and providing more descriptive information of *C. bovis.*

The present study demonstrates the localized response of the cardiac muscle around the parasite and the infiltration of the immune cells like macrophages, plasma, mast cells and lymphocytes in the areas around the *Cysticercus*. The severity of the histological lesions depends on the intensity of the inflammatory response [37]. The previous studies classify the lesions according to the severity of the immune response into different stages [38,39]. These stages start when the viable parasite is surrounded by a thin layer of the collagen fibers, then by the invasion of mononuclear inflammatory cells, and ending with the formation of granulomatous tissue with centers containing amorphous materials and the destructed parasite in a state of chronic infection. This occurs in agreement with the present results that show how some lesions that contain a layer of collagen fiber are infiltrated by the immune cells which are surrounded by the parasite and how other lesions had large number of the fibroblasts and dense layers of connective tissue. Furthermore, the current study uses immunohistochemical staining of Bax to demonstrate the apoptotic effect of the cysticercosis infection. The Bax protein is considered an apoptotic inducer, present in many tissues like hepatocyte, lymphocyte, and myocyte [40]. Apoptosis is considered the main defense action against intracellular infections [41]. The present study shows strong positive staining within the cardiac muscle that surrounds the cyst and in the inflammatory cells infiltrating the cyst, which is consistent with several previous studies [40,41].

Additionally, cysticercosis has various effects on the physiological condition of the heart depending on the severity and location of the infection. One of the most common effects of cysticercosis on the heart is inflammation and damage to the heart muscle, which can lead to myocarditis and cardiomyopathy [42]. The effect of cysticercosis on the physiological condition of the heart can be significant and potentially life-threatening [43]. To ascertain the impact of cysticercosis on the physiological state of sthe cardiac muscle, we have investigated various physiological parameters, such as the measurement of albumin, total proteins, troponin T, TAC, MDA, and LDH plasma levels. The present study shows insignificant changes in total protein and albumin levels that agrees with the previous study [44] reporting no changes in total proteins in naturally and experimentally infected cattle with *C. bovis*. Another study [45] finds significantly lowered serum total protein (*p* < 0.05) in infected animals (buffalo/cattle) that is attributed to decreased levels of albumin in comparison with a non-infected group. Others reports reveal a significant decrease in total serum proteins (*p* < 0.05) while there are high levels of albumin and globulin in infected cattle compared to the non-infected group [46]. These differences in the magnitude of the antibody response could be correlated with the number of cysts found at slaughter and the intensity of the infection (light, moderate, or severe) [44].

Cardiac troponin is a sensitive biomarker for the determination of minimal myocardial damage due to various conditions including trauma, inflammation, exposure to necrosis, and toxins [47,48]. Myocardial dysfunction could be detected through the blood levels of cardiac biomarkers such as troponin T, creatine kinase, LDH, and myoglobin. The present work reveals that there is an increase in the LDH and troponin T plasma levels of infected cattle as a marker of the muscle, cell, and tissue damage associated with many diseases and conditions [49]. The increasing level of serum creatine kinase and LDH during bovine cysticercosis could be due to the necrosis of muscular tissue that is enhanced by muscle fiber integration due to inflammatory cell infiltration because of degenerated or active metacestodes [44].

In addition, we found that the infected animals with *C. bovis* experience an increase in MDA levels and a decrease in TAC levels in plasma, reflecting the oxidative stress. The antioxidant enzymes, including TAC, superoxide dismutase, glutathione peroxidase, and catalase, play indispensable and fundamental roles in the protective capacity of biological systems against free radicals. Our results agree with a previous study [50] and reveal a statistically significant increase in MDA levels as a biomarker of lipid peroxidation, while there is a significant decrease in superoxide dismutase, glutathione peroxidase, and catalase in cattle tissues infected with cysticercosis compared with control samples. Moreover, *C. fasciolaris* infection leads to oxidation and apoptosis in rat livers as detected through antioxidant enzymes that show marked decline in the level of glutathione-S-transferase and glutathione activity, leading to the generation of reactive oxygen species [51]. Our results point out the pathological damage of *C. bovis* which is consistent with the idea that cysticercosis causes an oxidative stress due to the imbalance between free radical induction and the ability of the body to counteract or detoxify their harmful effects via neutralization by antioxidants, resulting in inflammation by cytokine infiltrations with damages to the host cells and tissue.

## 5. Conclusions

The current study reveals a set of epidemiological, morphological, and histopathological findings about the occurrence of bovine cardiac cysticercosis in Upper Egypt. It is important to note that bovine cysticercosis remains a public health concern in Egypt and further efforts are needed to sustain the decline in prevalence and prevent its spread. However, more research is needed to determine the epidemiological trend of *C. bovis* prevalence in Egypt over the years and the factors that influence its prevalence combined with the biomarkers associated with the infection.

## Figures and Tables

**Table 1:** Prevalence rate of *C. bovis* in examined slaughtered cattle in relation to breed, sex, and season.

N = 941	Positive *C. bovis* (*n* = 102)	*p* Value
Breed		<0.001 *
Native breed	102 (100%)	
Others	0 (0%)	
Sex		=0.006 *
Female	0 (0%)	
Male	102 (100%)	
Season		<0.001 **
Fall	4 (3.9%)	
Winter	7 (6.9%)	
Spring	12 (11.8%)	
Summer	79 (77.5%)	

(*) Fisher’s exact test was used to compare frequency between groups and (**) the Chi-square test was used to compare frequency between groups.

**Table 2:** The effect of *Cysticercus bovis* on plasma total proteins, albumin, MDA, TAC, troponin T, and LDH (*n* = 5 in each group).

Groups/ Parameters	Negative *Cysticercus bovis*	Positive *Cysticercus* *bovis*	*p* Value
Total protein (g/dL)	6.653 ± 0.0437	6.733 ± 0.1257	0.5803
Albumin (g/dL)	3.190 ± 0.0819	2.940 ± 0.2100	0.3295
MDA (nmol/mL)	1.943 ± 0.5261 ^a^	3.551 ± 0.1802 ^b^	0.0445
TAC (mM/L)	1.096 ± 0.0305 ^a^	0.953 ± 0.0195 ^b^	0.0168
Troponin T (pg/mL)	6.590 ± 0.1418 ^a^	8.267 ± 0.4153 ^b^	0.0188
LDH (U/L)	2247 ± 169.2 ^a^	2805 ± 102.3 ^b^	0.0479

Means ± SE, ^a, b^ the least square mean with an arrow is lacking a common superscript differ (*p* < 0.05).

## Data Availability

Data are contained within the article.
